# Dynamics of Tryptophan Metabolic Pathways in Human Placenta and Placental-Derived Cells: Effect of Gestation Age and Trophoblast Differentiation

**DOI:** 10.3389/fcell.2020.574034

**Published:** 2020-09-18

**Authors:** Rona Karahoda, Cilia Abad, Hana Horackova, Petr Kastner, Jonas Zaugg, Lukas Cerveny, Radim Kucera, Christiane Albrecht, Frantisek Staud

**Affiliations:** ^1^Department of Pharmacology and Toxicology, Faculty of Pharmacy in Hradec Kralove, Charles University, Hradec Kralove, Czechia; ^2^Department of Pharmaceutical Chemistry and Pharmaceutical Analysis, Faculty of Pharmacy in Hradec Kralove, Charles University, Hradec Kralove, Czechia; ^3^Institute of Biochemistry and Molecular Medicine, University of Bern, Bern, Switzerland; ^4^Swiss National Centre of Competence in Research (NCCR) TransCure, University of Bern, Bern, Switzerland

**Keywords:** fetal programming, trophoblast, tryptophan metabolism, placenta–brain axis, kynurenine pathway, serotonin pathway

## Abstract

L-Tryptophan is an essential amino acid and a precursor of several physiologically active metabolites. In the placenta, the serotonin and kynurenine metabolic pathways of tryptophan metabolism have been identified, giving rise to various molecules of neuroactive or immunoprotective properties, such as serotonin, melatonin, kynurenine, kynurenic acid, or quinolinic acid. Current literature suggests that optimal levels of these molecules in the fetoplacental unit are crucial for proper placenta functions, fetal development and programming. Placenta is a unique endocrine organ that, being equipped with a battery of biotransformation enzymes and transporters, precisely orchestrates homeostasis of tryptophan metabolic pathways. However, because pregnancy is a dynamic process and placental/fetal needs are continuously changing throughout gestation, placenta must adapt to these changes and ensure proper communication in the feto-placental unit. Therefore, in this study we investigated alterations of placental tryptophan metabolic pathways throughout gestation. Quantitative polymerase chain reaction (PCR) analysis of 21 selected genes was carried out in first trimester (*n* = 13) and term (*n* = 32) placentas. Heatmap analysis with hierarchical clustering revealed differential gene expression of serotonin and kynurenine pathways across gestation. Subsequently, digital droplet PCR, Western blot, and functional analyses of the rate-limiting enzymes suggest preferential serotonin synthesis early in pregnancy with a switch to kynurenine production toward term. Correspondingly, increased function and/or protein expression of serotonin degrading enzyme and transporters at term indicates efficient placental uptake and metabolic degradation of serotonin. Lastly, gene expression analysis in choriocarcinoma-derived cell lines (BeWo, BeWo b30, JEG-3) revealed dissimilar expression patterns and divergent effect of syncytialization compared to primary trophoblast cells isolated from human term placentas; these findings show that the commonly used *in vitro* placental models are not suitable to study placental handling of tryptophan. Altogether, our data provide the first comprehensive evidence of changes in placental homeostasis of tryptophan and its metabolites as a function of gestational age, which is critical for proper placental function and fetal development.

## Introduction

Placenta is a multifunctional organ providing the fetus with optimal conditions for its growth, development, and programming (Staud and Karahoda, 2018). As a continuously maturing organ, it undergoes structural (Kingdom et al., 2000), epigenetic, and transcriptomic (Uuskula et al., 2012; Cox et al., 2015) changes to adapt to its own as well as maternal and fetal demands. Correspondingly, a wide number of biological processes and molecular and metabolic pathways are differentially affected during gestation (Mikheev et al., 2008; Sitras et al., 2012).

Tryptophan (TRP) is an important amino acid necessary for protein synthesis as well as a precursor of several biologically active metabolites. During pregnancy, TRP and its metabolites are of crucial importance for placentation, fetal development, and immune regulation (Sedlmayr et al., 2014; Laurent et al., 2017). In the placenta, two main TRP metabolic pathways have been identified: the serotonin (5-HT) (Bonnin et al., 2011) and kynurenine (KYN) pathways (Sedlmayr et al., 2002; Goeden et al., 2017). The rate-limiting enzyme of the 5-HT pathway, tryptophan hydroxylase (TPH), gives rise to 5-HT, an important trophic factor early in gestation (Bonnin et al., 2011). Within the placenta a fraction of 5-HT is additionally metabolized to melatonin (Lanoix et al., 2008), which is involved in circadian rhythmicity, fetal growth, and placental function regulation (Iwasaki et al., 2005; Nagai et al., 2008; Seron-Ferre et al., 2012). Several studies have shown that maternal 5-HT also contributes to fetal 5-HT levels (Cote et al., 2007; Gleason et al., 2010; Muller et al., 2017). While early in pregnancy the fetus is dependent on placental/maternal 5-HT, from midgestation it synthesizes its own 5-HT from maternally derived TRP (Arevalo et al., 1991; Sano et al., 2016) suggesting that placental/maternal 5-HT is no longer needed. Indeed, in our latest study (Karahoda et al., 2020) we observed that at term, rat and human placenta does not provide 5-HT to the fetus; in contrast, it takes up fetal 5-HT across the basal membrane of the syncytiotrophoblast (STB) for subsequent degradation by monoamine oxidase-A (MAO-A). Together these findings indicate that placental handling of 5-HT changes throughout gestation.

The KYN pathway generally accounts for most of the TRP degrading activity via the rate-limiting enzymes, indoleamine 2,3-dioxygenase-1/2 (IDO1/2), and tryptophan 2,3-dioxygenase (TDO) (Sedlmayr et al., 2014). In the placenta, this pathway plays a crucial role in preventing fetal rejection by the maternal immune system (Munn et al., 1998). Extensive studies have been carried out to evaluate IDO1 expression/localization in the placenta, indicating that IDO1 expression/function increases during gestation (Sedlmayr et al., 2002; Ligam et al., 2005; Blaschitz et al., 2011; Murthi et al., 2017; Wakx et al., 2018), yet the exact localization in the placenta remains contradictory (Sedlmayr et al., 2014). Contrary to other studies (Sedlmayr et al., 2002; Honig et al., 2004; Kudo et al., 2004), it has been recently observed that IDO1 is not expressed in villous or extravillous trophoblast and the increasing IDO activity at term is exclusively due to expression in endothelial cells (playing a role in immunosuppression and placental tone relaxation) (Blaschitz et al., 2011). KYN is further metabolized to kynurenic acid (KYNA) and quinolinic acid (QUIN), which have neuroprotective and neurotoxic properties, respectively (Foster et al., 1984; Schwarcz et al., 2012). However, the importance of placental KYNA and QUIN remains to be fully elucidated. Recent studies in mouse term placenta report minimal placental contribution to fetal KYNA levels (Goeden et al., 2017; Notarangelo et al., 2019). Importantly, little is known about the effects of gestational age on expression and function of the enzymes down the KYN metabolic pathway, particularly those responsible for production of KYNA and QUIN.

Recently, the importance of gut microbiome metabolism of TRP for gut–brain axis has been described (Kaur et al., 2019; Gao et al., 2020). Similarly, placental metabolism of TRP might form a crucial component of the placenta–brain axis (Rosenfeld, 2020a,b). Considering the large spectrum of TRP metabolites and their roles in pregnancy, it is important to elucidate and understand the shifts in enzyme/transporter expression/activity occurring during gestation. Knowledge on the interplay between enzymes and transporters could provide a better understanding on the significance of a specific pathway at a certain point in pregnancy. Thus, in our study we investigated how advancing gestation affects expression and function of selected enzymes/transporters involved in placental homeostasis of TRP and its metabolites. In addition, we analyzed the effect of cell/trophoblast differentiation on gene expression patterns in isolated primary trophoblast cells and placenta-derived cell lines (BeWo, BeWo b30 clone, JEG-3) to assess their suitability for designated studies.

## Materials and Methods

### Chemicals and Reagents

Serotonin hydrochloride, L-Tryptophan, and phenelzine (MAO inhibitor) were purchased from Sigma–Aldrich (St. Louis, MO, United States). Forskolin (proliferation-activating agent) was obtained from Scintila, s.r.o. (Jihlava, CZ). Bicinchoninic acid assay (BCA assay) reagents were purchased from Thermo Fisher Scientific (Waltham, MA, United States). All other chemicals were of analytical grade.

### Human Placenta Sample Collection

First-trimester placentas (*n* = 13) were obtained after elective interruption of healthy pregnancy between 8 and 11 weeks of gestation. Term placentas (*n* = 32 for mRNA/protein/functional analysis and *n* = 5 for primary trophoblast isolation) were obtained from uncomplicated pregnancies at 38 to 40 weeks of gestation immediately after delivery. Samples were collected at the University Hospital in Hradec Kralove, Czech Republic or at the Division of Gynecology and Obstetrics, Lindenhofgruppe, Bern, Switzerland. All experiments were performed in accordance with the Declaration of Helsinki and human placenta samples were obtained upon women’s written informed consent and with the approval of the University Hospital Research Ethics Committee (201006 S15P) and Ethics Committee of the Canton of Bern (Basec No. 2016-00250).

### Choriocarcinoma-Derived Cell Cultures

The human choriocarcinoma-derived BeWo and JEG-3 cell lines were obtained from the European Cell Culture Collection (ECACC; Salisbury, Wiltshire, United Kingdom). BeWo cells were cultured in Ham F-12 medium supplemented with 10% fetal bovine serum (FBS), whereas JEG-3 cells were cultured in MEM medium supplemented with 10% FBS.

The human choriocarcinoma-derived BeWo b30 cell line (known to form integral monolayers) was obtained from Dr. A. Schwartz (Washington University, St. Louis, United States). Cells were cultured in Dulbecco modified eagle medium (high glucose) supplemented with 10% FBS.

All cell lines were cultivated without antibiotics at 37°C/5% CO_2_. For differentiation induction, BeWo b30 cell line was treated with 100 μM forskolin for 72 h with daily change of medium.

### Isolation and Characterization of Primary Trophoblast Cells

Villous cytotrophoblast cells (CTBs) were isolated from term placental tissue by enzymatic digestion and Percoll gradient separation, as previously described, with minor modifications (Kallol et al., 2018). In brief, approximately 50 g of villous tissue was washed in 0.9% NaCl (Sigma–Aldrich) four times for 5 min. Thereafter, the tissue was minced and digested three times with 0.25% trypsin (Sigma–Aldrich) and 300 IU/mL deoxyribonuclease I (Sigma–Aldrich) at 37°C (20 min each). The cell suspension was filtered and overlaid on FBS (Seraglob, Switzerland). After centrifugation at 1,000 × g for 15 min at 10°C, the cell pellet was collected in Dulbecco modified eagle medium (high glucose) basic medium (without FBS) and filtered through 100-μm strainer (BD Biosciences, San Jose, CA, United States). Next, cells were overlaid on a discontinuous Percoll^®^ (Sigma–Aldrich) density gradient. After centrifugation, CTBs were located at the layer corresponding to 1.046–1.065 g/mL (35–50%) density (Petroff et al., 2006). The isolated CTBs were cultured at a density of 1 × 10^6^ cells/cm^2^ in 6-well CellBIND^®^ plates (Corning, New York, NY, United States) in Dulbecco modified eagle medium (high glucose) supplemented with 10% FBS and 1% antibiotic–antimitotic (Thermo Fisher Scientific). Cells were cultured for 12 h (CTB stage) or 72 h (STB stage).

### Flow Cytometry Analysis of Primary Trophoblast Cell Purity

The purity of the isolated trophoblast (PHT) cells was evaluated by staining with specific cell markers followed by flow cytometry analysis as previously described with minor modifications (Kallol et al., 2018). Cells were grown on CellBIND^®^ plates, detached by Accutase^®^ (Sigma–Aldrich), and fixed in 4% formaldehyde (Thermo Fisher Scientific) for 10 min on ice. After washing with Dulbecco phosphate-buffered saline (DPBS; Sigma–Aldrich) cells were centrifuged at 200 *g* for 10 min at 4°C and then permeabilized with 0.5% Tween-20 (wt/vol) (Sigma–Aldrich) in DPBS for 15 min at room temperature. For evaluation of cell purity, dual staining of CTB and STB with directly labeled antibodies (Novus Biologicals, CO, United States) prepared in staining buffer (5% FBS, 0.1% Tween-20 (wt/vol) in DPBS), was performed. Antibody cocktails comprised (1) anti–cytokeratin 7 (CK-7; AF 488^®^) plus anti-vimentin (Vim; AF 647^®^); (2) anti–E-cadherin (E-cad; AF 488^®^) plus anti-von Willebrand Factor (vWF; AF 647^®^). Cells were incubated with the respective antibody cocktail for 45 min on ice, followed by two times washing in DPBS (1 min each). After centrifugation at 200 *g* for 10 min at 4°C, pelleted cells were suspended in DPBS and acquired by flow cytometry (BD FACS LSRII; BD Biosciences). Data acquisition and analysis for each staining were based on at least 10,000 events and performed by using BD FACSDiva^TM^ (BD Biosciences) and FlowJo^®^ software version 10 (FlowJo LLC, Ashland, OR, United States). Because CTB and STB are epithelial cells, staining for CK-7 and E-cad served as positive cell markers (Maldonado-Estrada et al., 2004; Li and Schust, 2015). Anti-vim, known to predominantly stain mesenchymal cells, fibroblast, and stromal cells and anti-vWF staining endothelial cells (Zanetta et al., 2000; Maldonado-Estrada et al., 2004; Li and Schust, 2015) served to quantify potential cellular contaminations by other cell types.

### RNA Isolation, Reverse Transcription, and Quantitative Polymerase Chain Reaction Analysis

RNA isolation was performed using Tri Reagent (Molecular Research Centre, Cincinnati, OH, United States) or Trizol (Invitrogen, Carlsbad, CA, United States) according to the manufacturer’s instructions. RNA concentration was calculated by measuring absorbance (A) at 260 nm and purity by the A260/280 and A260/230 ratios measured on NanoDrop^TM^ 1000 Spectrophotometer (Thermo Fisher Scientific). RNA integrity was confirmed by electrophoresis on a 1.5% agarose gel. 1 μg of total RNA was reversely transcribed to cDNA in a total volume of 20 μL using the iScript cDNA Synthesis Kit (Bio-Rad, Hercules, CA, United States) on a Bio-Rad T100^TM^ Thermal Cycler; for primary trophoblast cells using oligo(dT)15 primers and GoScript^TM^ Reverse Transcriptase System (Promega, Madison, WI, United States) according to the manufacturer’s instructions.

cDNA (12.5 ng/μL) was amplified in QuantStudio^TM^ 6 (Thermo Fisher Scientific) using the TaqMan^®^ Universal Master Mix II without UNG (Thermo Fisher Scientific) and predesigned TaqMan^®^ Real Time Expression polymerase chain reaction (PCR) assays (listed in Supplementary Table 1, Additional File 1). PCR analysis was run in 5 μL volume, in 384-well plate format. Each sample was amplified in triplicate, following the thermal conditions according to the manufacturer’s instructions.

Prior to quantitative analysis, we evaluated several reference genes for their stable expression during gestation/upon differentiation. Target gene expression in choriocarcinoma-derived cell cultures and primary trophoblast cells was normalized against the predesigned reference gene tyrosine 3-monooxygenase/tryptophan 5-monooxygenase activation protein zeta (*YWHAZ*; Thermo Fisher Scientific) using the 2^ΔΔCt^ method, whereby ΔCt = Ct_ref_ – Ct_target_ and ΔΔCt = ΔCt_differentiated_ – ΔCt_undifferentiated_. On the other hand, gene expression of target genes in human placenta samples was normalized against the predesigned reference gene TATA-box binding protein (*TBP*; Thermo Fisher Scientific) using the ΔCt method, whereby ΔCt = Ct_ref_ – Ct_target_. These values were used to generate a gene expression heat map, through the freely available web server Heatmapper^1^ (Babicki et al., 2016). Hierarchical clustering (Average linkage, Euclidean distance) was applied to group samples with similar expression levels. The scatter plot was constructed in GraphPad Prism 8.3.1 software (GraphPad Software, Inc., San Diego, CA, United States) using the average 2^Δ*Ct*^ values for first-trimester and term placentas.

### Droplet Digital PCR Assay

Absolute quantification of *SLC6A4*, *SLC22A3*, *MAO-A*, *TPH1*, *TPH2*, *IDO1*, and *IDO2* in human first-trimester and term placentas was performed using duplex droplet digital PCR (ddPCR) analysis, as described previously (Karahoda et al., 2020). Briefly, the duplex reaction mixture consisted of 10 μL of ddPCR^TM^ Supermix for Probes (Bio-Rad), 1 μL of each of the predesigned probe assays (target – FAM and reference – HEX) (listed in Supplementary Table 1, Additional File 1), and 0.5 μL of cDNA (50 ng/μL), in a total volume of 20 μL. Droplets were generated using QX200 Droplet Generator and subsequently amplified to end-point using T100^TM^ Thermal Cycler following the thermal conditions recommended by the manufacturer. Droplet counting was performed in QX200^TM^ Droplet Reader and the concentration of the target gene was calculated using the QuantaSoft^TM^ Software. For final data evaluation, only wells in which the number of droplets obtained was higher than 13,000 were used. Expression levels are reported in number of transcripts/ng of transcribed RNA. The QX200^TM^ Droplet Digital^TM^ PCR System, T100^TM^ Thermal Cycler, and all consumables and reagents were obtained from Bio-Rad (unless otherwise stated).

### Preparation of Human Placenta Homogenates

Human first-trimester and term placentas were washed with 0.9% NaCl at 4°C. After weighing and cleaning, the decidua and the chorionic plate were removed, and the placentas were cut in small pieces and homogenized at 4°C in a buffer containing 50 mM Tris-HEPES (pH 7.2), 5 mM EGTA, 5 mM EDTA, 1 mM phenylmethylsulfonyl fluoride, and 250 mM sucrose. The homogenates were filtered through gauze and centrifuged at 15,000 *g* for 10 min. The supernatant was collected and stored in the freezer at −80°C until use. Protein concentration was determined using the BCA protein assay kit.

### Western Blot Analysis

Aliquots of placenta homogenates (30 μg total protein) were mixed with loading buffer under reducing conditions (Laemmli, 1970), heated at 96°C for 5 min, and separated by SDS-PAGE on polyacrylamide gels (10% for SLC6A4, SLC22A3, and MAO-A; 15% for IDO and TPH). Electrophoresis was performed at 150 V and proteins were transferred to polyvinylidene fluoride (PVDF) membranes (SERVA, Heidelberg, DE). The membranes were blocked in 20 mM Tris-HCl pH 7.6, 150 mM NaCl, 0.1% Tween 20 (TBS-T) containing 5% non-fat milk for 1 h at room temperature and washed with TBS-T buffer. Incubation with primary antibodies against SLC6A4, SLC22A3, MAO-A, IDO, and TPH (listed in Supplementary Table 2, Additional File 1) was performed overnight at 4°C. After washing with TBS-T buffer, the membranes were incubated with a specific secondary antibody (listed in Supplementary Table 2, Additional File 1) for 1 h at room temperature. Membranes were developed using Chemiluminescence HRP Substrate Kit (SERVA Light Vega). Band intensity was visualized and quantified by densitometric analysis using ChemiDoc MP, Imaging system^TM^ (Bio-Rad). To ensure equal loading of proteins, membranes were probed for β-actin and specific secondary antibody (listed in Supplementary Table 2, Additional File 1).

### 5-HT Metabolism by MAO-A in Human Placenta Homogenates

MAO-A activity was determined by the method of Carrasco et al. (2000). Briefly, 180 μL placenta homogenate (1.5–2 mg/mL) was pre-incubated with or without MAO-A inhibitor, phenelzine (100 μM) for 5 min at 37°C, and then the reaction was initiated by incubation with 20 μL of 5-HT (0.5 mM) for an indicated time period. The reaction was stopped by adding 40 μL of HClO_4_ (3.4 M) and placed on ice for 5 min. Samples were centrifuged at 5,000 *g* for 10 min, and the supernatant was used for 5-HT determination by high-performance liquid chromatography (HPLC).

### IDO Enzymatic Activity

IDO activity was determined by the method of Takikawa et al. (1988). The incubation media (50 mM potassium phosphate buffer pH 6.5, 20 mM ascorbate, 0.01 mM methylene blue, 100 units/mL catalase) was pre-incubated for 5 min at 37°C, with or without 0.4 mM TRP. The reaction was initiated by adding the placenta homogenate and terminated after 30 min with 200 μL of trichloroacetic acid 30%. Samples were further incubated for an additional 30 min at 50°C to assure complete hydrolysis of N-formyl KYN to KYN. The reaction mixture was then centrifuged for 20 min at 3,000 *g*, 20°C and supernatant was collected for HPLC measurement of KYN. The IDO enzymatic activity was calculated as the difference between the amount of KYN produced in the media with and without TRP. The results are expressed as nmol KYN/μg protein per min.

### TPH Enzymatic Activity

TPH enzymatic activity was determined by the method by Goeden et al. (2016). Human placenta homogenates were supplemented with 1 mM dithiothreitol as a reducing agent ensuring complete enzymatic activity (Fitzpatrick, 1999). The enzymatic reaction was carried out at 37°C, pH 7.5, with ∼1.5 to 2 mg protein/mL. The incubation media contained (final concentrations): 50 mM Tris buffer, 1 mM EGTA, 100 units/mL catalase, 0.1 mM ammonium iron (II) sulfate, 0.1 mM tetrahydrobiopterin (BH4, a cofactor required for TPH activity), either in the absence or in the presence of 0.25 mM TRP. Briefly, placenta homogenate was incubated with the incubation media for 30 min at 37°C. Reaction was terminated by adding 200 μL of HClO_4_ with 100 μM EDTA. Samples were incubated on ice for 15 min for complete protein denaturation and then centrifuged for 15 min at 21,000 *g*. Supernatants were collected for determination by HPLC of 5-hydroxytryptophan (5-OH-TRP), a metabolic intermediate in 5-HT synthesis. The results were calculated as the difference between the amount of 5-OH-TRP liberated in samples with and without TRP and are expressed as nmol 5-OH-TRP/μg protein per min.

### HPLC Analysis of TRP Metabolites in Placental Homogenates

The HPLC analyses were performed using Shimadzu LC20 Performance HPLC chromatograph (Shimadzu, Kyoto, Japan) equipped with UV and fluorescence detector. For simultaneous chromatographic separation of all tested compounds, Phenomenex Kinetex 5 μm EVO C18 100 A 150 × 3 mm with a guard column was used. An isocratic elution, at a flow rate of 0.5 mL/min, was performed with mobile phase consisting of 0.1 M acetic acid, pH 4.5 (adjusted with NaOH), and methanol 97 + 3. All analytes were eluted within 8.5 min.

Excitation and emission wavelengths of fluorescence detector were set for individual compounds: 275/333 nm for 5-OH-TRP from 0 to 3.1 min and 280/334 nm for 5-HT and TRP from 3.1 min. KYN was detected by UV detection with wavelength set to 369 nm. Additionally, in cases of TRP concentrations higher than the range of fluorescence detection, UV detection was used with wavelength set to 300 nm.

### Statistical Analysis

Quantitative PCR (qPCR) results were assessed using Mann–Whitney tests. ddPCR analyses, protein expression, and functional studies were evaluated using unpaired *t*-test. All statistical analyses were implemented in GraphPad Prism 8.3.1 software (GraphPad Software, Inc.). Asterisks in the figures indicate significance levels: ^∗^*p* ≤ 0.05, ^∗∗^*p* ≤ 0.01, and ^∗∗∗^*p* ≤ 0.001.

## Results

### Clinical Characteristics

**FIGURE 1:**
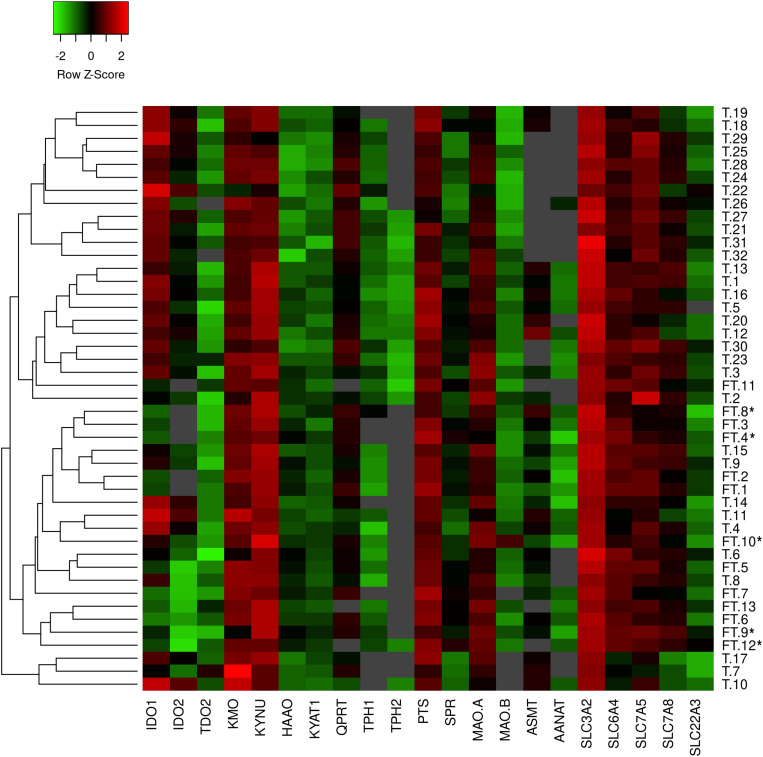
Heatmap generated from qPCR gene expression analysis of TRP pathway in human placenta. Expression of 21 key transporters/enzymes was analyzed in first trimester (FT; *n* = 13) and term (T; *n* = 32) human placenta samples. Hierarchical clustering (average linkage, Euclidean distance) was applied to group samples with similar expression levels, emphasizing the overall changed expression during gestation. The color intensity indicates expression levels; red: up-regulation, green: down-regulation, gray: not detected. *Placentas from smokers.

Characteristics of the first-trimester and term pregnancies are listed in Table 1. No statistical differences were found in the mean maternal age and maternal BMI before pregnancy between the two groups. In the term group (*n* = 32) only healthy, non-medicated and non-smoking mothers were included. On the other hand, 5 of 13 women from the first trimester samples were smokers (frequency: three women < 10 cigarettes/day; two women > 10 cigarettes/day). Placenta samples from these women were included in the PCR analysis only if no association between smoking and gene expression was found. These samples have been marked with an asterisks (^∗^) in the heatmap representing gene expression data (Figure 1).

**TABLE 1:** Clinical characteristics of first-trimester and term pregnancies involved in the study.

**Parameter**	**First trimester (*n* = 13)**	**Term (*n* = 37)**
Maternal age (years)	27.91 ± 8.04	32.24 ± 5.07
Gestational age (weeks)	9.62 ± 1.19	39.57 ± 1.03
Smoking (Y:N)	5:8	0:37
Maternal BMI before pregnancy (kg m^–2^)	24.48 ± 3.31	24.67 ± 4.59
Maternal BMI at delivery (kg m^–2^)	NA	29.28 ± 4.30
Labor (NSVD:CS)	NA	21:16
Birth weight (kg)	NA	3.30 ± 0.44
Birth height (cm)	NA	49.94 ± 1.93
Fetal sex (M:F)	NA	20:17

*Parameters are expressed as mean ± SD. CS, cesarean section; F, female; M, male; NA, not applicable; N, no; NSVD, normal spontaneous vaginal delivery; Y, yes.*

### Relative Gene Expression Analysis of TRP Metabolic Pathways in Human First-Trimester and Term Placenta

Human placenta fully expresses the enzymatic machinery for TRP metabolism to 5-HT and KYN pathways. A heatmap with hierarchical clustering revealed sample distribution into three main clusters, whereby the first-trimester placentas were clustered predominantly into one cluster, whereas term placenta samples were distributed into the remaining two clusters (Figure 1); the pattern of distribution indicated differential expression of the pathways across gestation. Subsequently, individual analysis of genes in first-trimester and term placentas showed several enzymes/transporters to be significantly up- or down-regulated at term.

**FIGURE 2:**
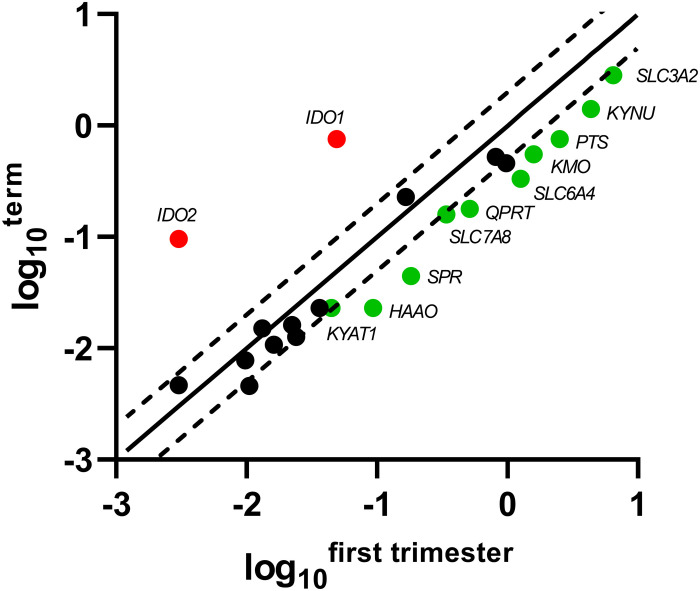
Scatter plot of the TRP pathway enzymes/transporters showing significant changes in gene expression during gestation. Log_10_ of each gene-specific 2^Δ*Ct*^ in the first-trimester placenta is plotted against those of term placenta. The central diagonal line shows unchanged gene expression, whereas the dotted lines depict the upper and lower limit of the threshold fold regulation, which was set to 2. Data were further evaluated using non-parametric Mann–Whitney test on ΔCt values, and those that exceed the threshold fold regulation and were statistically significant (*p* ≤ 0.05) are named and shown in color: red – up-regulation, green – down-regulation.

Of the 5-HT pathway, gene expression of the main enzymes (*PTS*, *SPR*) responsible for BH4 production, a co-factor necessary for TPH function, were found to be significantly decreased at term, compared with first-trimester placenta. On the other hand, the rate-limiting enzymes of the KYN pathway, *IDO1/2*, were negligibly expressed in the first-trimester placenta, whereas we observed significant expression at term. Interestingly, the subsequent enzymes of the KYN pathway (specifically *KMO*, *KYNU*, *HAAO*, *KYAT1*, *QPRT*) were found to be expressed in lower amounts at term, compared with first-trimester placenta. Lastly, of the transport proteins tested, *SLC3A2*, *SLC6A4*, and *SLC7A8* revealed higher expression in the first-trimester placenta. Scatter plots of the log_10_-expression in first-trimester and term placenta were used to display the data and visualize the gene expression differences (Figure 2).

### ddPCR Quantification of TPH1, TPH2, MAO-A, IDO1, IDO2, SLC6A4, and SLC22A3 Transcripts

**FIGURE 3:**
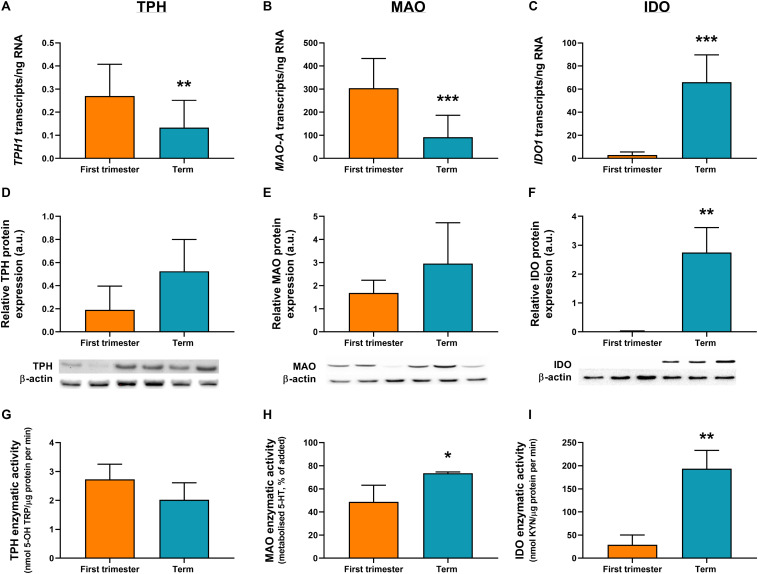
Gene/protein expression and functional analyses of the rate-limiting enzymes of 5-HT and KYN pathways. Expression and function of tryptophan hydroxylase-1 (*TPH1*/TPH1), monoamine oxidase-A (*MAO-A*/MAO-A), and indoleamine 2,3-dioxygenase-1 (*IDO1*/IDO1) between the first-trimester and term human placenta was analyzed by digital droplet PCR **(A–C)**, Western blot **(D–F)**, and enzymatic activity **(G–I)** analyses. Protein expression was normalized to β-actin as a loading control; representative immunoblots for target proteins and β-actin are shown. For ddPCR analysis *n* = 13 (first trimester) and *n* = 25 (term placentas); for protein/functional analysis *n* = 3 for each. Data are presented as mean ± SD and statistical significance was evaluated using unpaired *t*-test; *p ≤ 0.05, **p ≤ 0.01, ***p ≤ 0.001.

**FIGURE 4:**
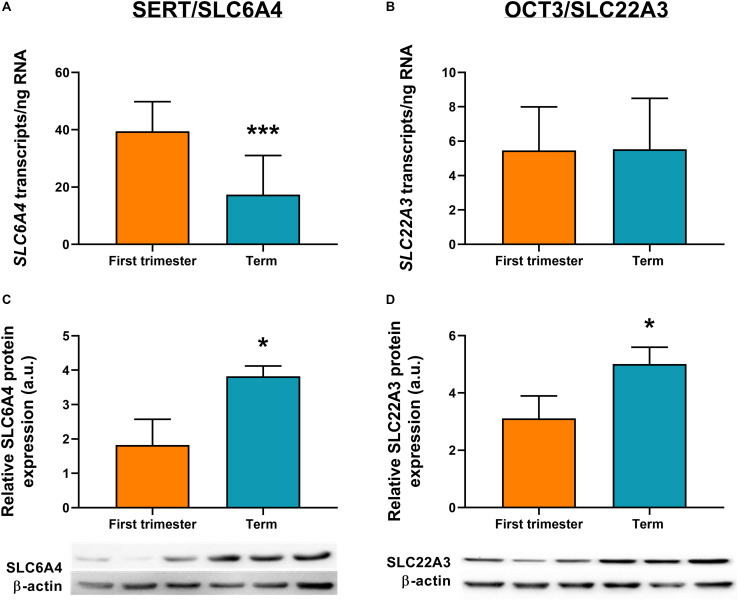
Gene and protein expression of the main transporters of 5-HT pathway. Comparison of gene and protein expression of 5-HT transporter (*SLC6A4*/SERT) and organic cation transporter 3 (*SLC22A3*/OCT3) between the first-trimester and term human placenta was analyzed by digital droplet PCR **(A,B)** and Western blot **(C,D)**. Protein expression was normalized to β-actin as a loading control; representative immunoblots for target proteins and β-actin are shown. For ddPCR analysis *n* = 13 (first trimester) and *n* = 25 (term placentas); for protein analysis *n* = 3 for each. Data are presented as mean ± SD and statistical significance was evaluated using unpaired *t*-test; *p ≤ 0.05, ***p ≤ 0.001.

ddPCR analysis was conducted in 13 first-trimester and 25 term placentas for absolute quantification of transcripts of the rate-limiting enzymes and main transporters of 5-HT and KYN pathways in the human placenta. We observed statistically significant down-regulation of *TPH1* (Figure 3A), *MAO-A* (Figure 3B), and *SLC6A4* (Figure 4A) at term, whereas *IDO1* (Figure 3C) and *IDO2* (Supplementary Figure 1B, Additional File 1) gene expression was up-regulated. *SLC22A3* (Figure 4B) and *TPH2* (Supplementary Figure 1A, Additional File 1) expression remained unchanged during gestation.

While human placenta expresses both isoforms (1 and 2) of *TPH* and *IDO* (Sedlmayr et al., 2014; Laurent et al., 2017; Ranzil et al., 2019), we found that *TPH1* and *IDO1* are predominant throughout gestation. Specifically, *IDO1* (Figure 3C) levels in the first-trimester and term placenta exceeded those of *IDO2* (Supplementary Figure 1B, Additional File 1) by 30- and 11-fold, respectively. On the other hand, *TPH1* (Figure 3A) showed three to six times higher expression compared to *TPH2* (Supplementary Figure 1A, Additional File 1), during gestation.

Moreover, we observed that *MAO-A* transcripts outnumbered those of *TPH1* transcripts by more than 1,000-fold in the first trimester and almost 700-fold at term (Figures 3A,B). On the other hand, while *TPH1* expression remained unchanged during pregnancy, we observed a 20-fold increase in *IDO1* transcripts at term (Figure 3C), suggesting a shift/preferential TRP metabolism toward the KYN pathway at term.

### Protein Analysis

To investigate the expression at protein level, western blot analysis using specific antibodies for TPH, MAO, IDO, SLC6A4, and SLC22A3 was performed in homogenates from first-trimester (*n* = 3) and term (*n* = 3) human placenta samples. We observed no difference in MAO-A and TPH1 protein expression during gestation (Figures 3D,E). On the other hand, IDO1 protein band (45 kDa) was clearly detected in term placenta homogenates, whereas it was not visible in first trimester samples (Figure 3F), indicating that IDO1 protein is not expressed at early stages of pregnancy. As for the transport proteins, both SLC6A4 (Figure 4C) and SLC22A3 (Figure 4D) showed a significantly increased protein expression in term placentas compared with the first trimester ones.

### Functional Analysis

#### TPH Activity

TPH activity was evaluated in first-trimester and term placenta homogenates, using TRP as a substrate and measuring the production of 5-OH TRP. As shown in Figure 3G, TPH activity ranged between 2 and 3 nmol 5-OH TRP/μg protein per min, and it was not affected by gestational age.

#### MAO Activity

MAO enzymatic activity was determined in first-trimester and term placenta homogenates by measuring the amount of 5-HT metabolized in the placental homogenate after 60 min of incubation with 5-HT. As shown in Figure 3H, after 60 min of incubation, 45% of 5-HT was metabolized by first-trimester placenta and nearly 75% by term placenta. Metabolism of 5-HT was completely inhibited by addition of phenelzine (100 μM) (Karahoda et al., 2020), indicating that 5-HT was metabolized specifically by MAO. These data suggest that placental metabolism of 5-HT increases towards the end of pregnancy.

#### IDO Activity

IDO activity in first-trimester and term placentas was evaluated using TRP as a substrate. IDO activity showed a significant increase during human pregnancy, with levels as low as 29 nmol KYN/μg protein per min (±17.3) in the first-trimester placenta to 7-fold higher activity at term (193 ± 32.3 nmol KYN/μg protein per min) (Figure 3I).

### Gene Expression of TRP Metabolic Pathways in Placental-Derived Cells

**FIGURE 5:**
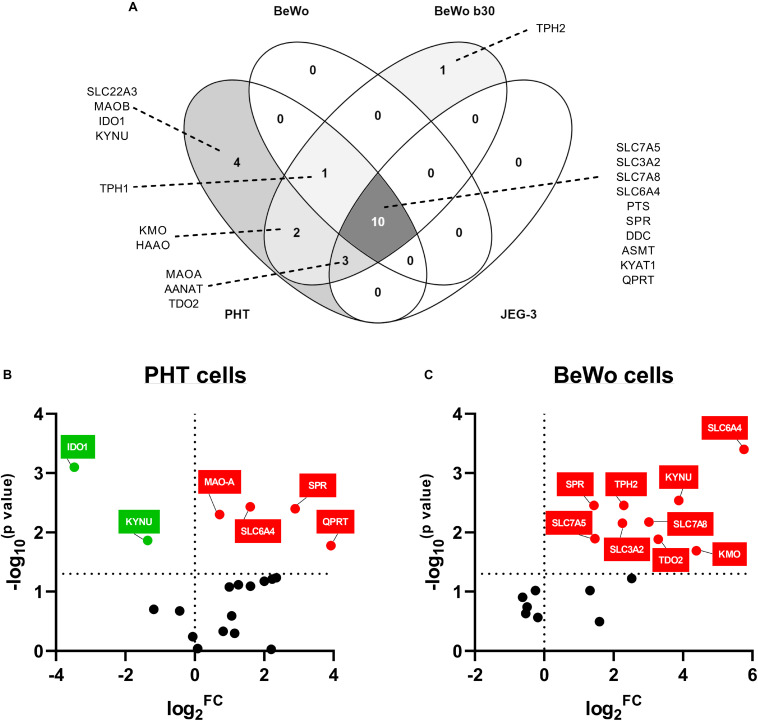
Gene expression of TRP pathway-related enzymes and transporters in placental-derived cells. Placental-derived cells were obtained from three different passages of BeWo, BeWo b30, and JEG cells and five isolations of primary human trophoblast cells (PHT). **(A)** Venn diagram of the overlap in gene expression among four cell lines. PHT and BeWo b30 cells share the highest gene homology, with the PHT cells additionally expressing four important genes, including the rate-limiting enzyme, *IDO1*, and the polyspecific organic transporter, *SLC22A3*. Subsequently, effect of differentiation in these two cell lines was tested, revealing dissimilarity between spontaneous syncytialization **(B)** and forskolin-induced differentiation **(C)**. Data are visualized as volcano plots of fold changed expression in the differentiated cells, compared to the undifferentiated counterpart. Color indicates statistically significant changes: red – up-regulation, green – down-regulation. Statistical significance was evaluated using Mann–Whitney test.

Choriocarcinoma-derived cell lines (BeWo, BeWob30, JEG-3) and PHT cells isolated from human term placentas, were analyzed for expression of the main enzymes/transporters of the TRP pathway. Out of 21 genes tested, only 10 were found to be co-expressed in all cell types, with the PHT and BeWo b30 cells showing the highest similarity in gene expression (Figure 5A). Nonetheless, none of the choriocarcinoma-derived cell lines expressed the rate-limiting enzyme of the KYN pathway, *IDO1* or the 5-HT uptake transporter (OCT3*/SLC22A3*), making the PHT cells the only suitable *in vitro* model for studies of the respective pathways.

### Effect of Differentiation on Gene Expression Profile in BeWo Cells and Human Primary Trophoblast Cells

Since the PHT and BeWo b30 cells shared the highest homology in genes expressed at mRNA level, we further studied the effect of differentiation in these cells. While CTB spontaneously syncytialized within 48–72 h, BeWo cell differentiation was induced with forskolin over a 72-h period. Only two of the tested genes (*SLC6A4* and *SPR*), revealed similar gene expression changes, specifically up-regulation, in BeWo and PHT cells upon differentiation. However, altogether, we observed that the differentiation process in BeWo cells affects the gene expression of TRP pathway enzymes/transporters in a more profound manner than in PHT cells (Figures 5B,C). Specifically, in PTH cells *IDO1* and *KYNU* were found to be down-regulated, whereas *MAO-A* and *QPRT* up-regulated (Figure 5B). Conversely, in BeWo cells we observed up-regulation for the following genes: *TPH2*, *KYNU*, *KMO*, *TDO2*, *SLC3A2*, *SLC7A5*, and *SLC7A8* (Figure 5C).

## Discussion

In our recent study (Karahoda et al., 2020) we demonstrated that the term placenta no longer provides 5-HT to the fetus. In contrast, it extracts it from the fetal circulation via OCT3-mediated process for subsequent degradation by MAO-A. We thus hypothesized that fetoplacental homeostasis of TRP and 5-HT changes throughout gestation. In this follow up study, we investigated the gene expression of 16 enzymes and 5 transporters involved in the metabolism and transport of TRP and its metabolites in human placenta (first trimester and term) and placental-derived cells. Moreover, using a combination of gene/protein expression and functional analyses, we further report that the key enzymes and transporters involved in placental homeostasis of TRP, 5-HT, and KYN exhibit changes throughout gestation, likely as a result of adaptation to meet different placental/fetal needs with time.

Placental expression of several enzymes involved in TRP metabolism has been investigated before (Ligam et al., 2005; Lanoix et al., 2008; Bonnin et al., 2011; Laurent et al., 2017) and related to pathological conditions such as fetal growth restriction and preeclampsia (Carrasco et al., 2000; Murthi et al., 2017; Ranzil et al., 2019). Nevertheless, comprehensive characterization of the metabolic pathways in human placenta and physiological changes that may occur during gestation are poorly understood. As TRP is an essential amino acid, the placenta and fetus are dependent on maternal intake and placental transport from the maternal to fetal circulation. Therefore, apart from the metabolizing enzymes, it is also important to study the expression and function of placenta membrane transporters responsible for fetoplacental handling of TRP and its metabolites.

TRP is a substrate of L-type amino acid transporter-1 (LAT1/*SLC7A5*) on the maternal-facing membrane and L-type amino acid transporter-2 (LAT2/*SLC7A8*) on both maternal- and fetal-facing membranes; LAT1/2 functional activity is dependent on heterodimerization with the 4F2 heavy chain (*SLC3A2*) (Gaccioli et al., 2015). We observed that placental expression of *SLC3A2* and *SLC7A8* is down-regulated at term, which contrasts with a recent examination of publicly available gene expression array data, reporting no change at any stage of pregnancy for these transporters (Simner et al., 2017). However, it should be noted that the small sample size (*n* = 4 per gestational age) compared with our cohort (*n* = 13 for first-trimester and *n* = 32 for term placenta) could account for the different outcomes.

TRP metabolism to 5-HT is mediated by TPH, whose activity depends on tetrahydrobiopterin (BH4) as a cofactor (Mckinney et al., 2005). Two mechanisms of BH4 synthesis in the human placenta have been suggested, *de novo* synthesis and/or salvage pathway (Iwanaga et al., 2004). We observed that the expression of 6-pyruvoyltetrahydropterin synthase (PTS), involved in *de novo* synthesis, and of sepiapterin reductase (SPR), involved in both pathways, decreased significantly at term, which corresponds nicely with previous reports on decreased SPR activity with increasing gestational age (Iwanaga et al., 2004). With the importance of BH4 as a cofactor for endothelial nitric oxide synthase (necessary for nitric oxide production), we speculate that decreasing SPR expression and activity at term may decrease the availability of BH4 for TPH activity, thus 5-HT synthesis at term.

Interestingly, we demonstrate that the first-trimester placentas show preferential expression of downstream enzymes of the KYN pathway, specifically *KMO*, *KYNU*, *HAAO*, and *QPRT* [involved in generation of 3-hydroxy-L-KYN (3-HK), 3-hydroxy anthranilic acid (3-HAA), and QUIN] and *KYAT1* (involved in generation of KYNA). This was unexpected because the rate-limiting enzyme IDO1 is, in contrast, only modestly expressed in the first-trimester placenta. While in 1998, Munn et al. (1998) suggested IDO1-based suppression of immune reactions to mediate fetomaternal tolerance, in a follow-up study, they reported that pregnancy success rate is not affected in the IDO-deficient mouse model (Baban et al., 2004). The authors proposed involvement of alternative processes, such as TDO, which may compensate for IDO activity when low or absent. In our study, we observed that *TDO* expression, although at relatively low levels, remains stable throughout gestation. Our results thus support a concept proposed by Badawy (2015) in which TRP degradation in early-to-mid pregnancy is catalyzed by TDO, with IDO gaining a partial/transient role in midgestation. We speculate that in the first trimester, KYN synthesis via TDO serves mainly as a precursor of 3-HK, 3-HAA, QUIN, and KYNA. QUIN is important for NAD^+^ synthesis, necessary for numerous redox reactions and DNA repair. Similarly, 3-HK and 3-HAA are important metabolites with antioxidant and immunosuppressive properties. Lastly, KYNA, apart from its immunosuppressive function, plays a role in neuroprotection, probably through its action on the NDMA receptor (Foster et al., 1984). In accordance with our qPCR data, recent studies in mouse placenta showed limited placental KYNA synthesis at term (Goeden et al., 2017; Notarangelo et al., 2019). Thus, we believe that the impact of these metabolites may be of higher importance in the first trimester when the pro-inflammatory environment is less pronounced (Hannan et al., 2014; Holtan et al., 2015). On the other hand, the significant increase in IDO1 at term could account for high KYN production involved in the immune related activities. Indeed, this concept was previously discussed by Badawy who suggests preferential TRP utilization for protein, 5-HT and NAD^+^ synthesis in early pregnancy (Badawy, 2015).

Therefore, we investigated the expression profiles and metabolic activity of the rate-limiting enzymes of the 5-HT and KYN pathways, TPH and IDO, respectively, early in pregnancy and at term. Our results indicate that during the first trimester, placenta may preferentially metabolize TRP to 5-HT, an important trophic factor for fetal development. Indeed, Bonnin et al. (2011) showed that placental 5-HT synthesis occurs as early as E10.5 in mice and week 11 in humans. It is during this period when the fetus is not capable of synthesizing 5-HT, yet serotonergic neurons and receptors have been identified (Bonnin and Levitt, 2011). As the immature fetal blood–brain barrier is not fully functional (Daneman et al., 2010), it has been well-established that the placenta serves as the main source of fetal 5-HT in early gestation (Bonnin and Levitt, 2011; Bonnin et al., 2011). However, later in gestation, the fetus gains the capability of 5-HT synthesis (Arevalo et al., 1991; Sano et al., 2016). With increased IDO expression/activity at term, also reported before (Blaschitz et al., 2011; Murthi et al., 2017; Wakx et al., 2018), it seems plausible that at later stages of gestation, TRP is preferentially utilized for KYN production.

In our previous study (Karahoda et al., 2020) we described the importance of membrane transporters (SERT/OCT3) and metabolizing enzyme (MAO-A) for placental 5-HT homeostasis at term. Here we reveal that, at protein levels, both transporters and the metabolizing enzyme are up-regulated at term. Increased OCT3 protein expression in human term placenta was also described by Lee et al. (2013). These findings suggest that towards term, placental capacity to take up 5-HT from both maternal and fetal circulations increases. The parallel increase in MAO-A expression and activity toward term strengthens our hypothesis that an orchestration between SERT, OCT3, and MAO-A activity serves as a 5-HT detoxification mechanism, protecting the term placenta and the fetus from high 5-HT circulating levels.

*In vitro* cell-based approaches (e.g., BeWo, BeWo b30, and JEG-3) are often applied as alternative methods to investigate placental physiology. However, these cells are derived from first trimester choriocarcinoma and, correspondingly, we show that gene expression of TRP metabolic pathways differs largely from that of primary trophoblast cells isolated from human term placenta. Specifically, BeWo and JEG-3 cells lack expression of crucial proteins, *IDO1* and *SLC22A3*; lack of *IDO1* in BeWo cells was also reported before (Entrican et al., 2002). In contrast, isolated PHT cells show expression pattern similar to that of term placenta. Another advantage of isolated PHT cells is their spontaneous fusion in culture to form the syncytium (Huang et al., 2016) while in BeWo cells, syncytialization must be provoked by modulators of cAMP metabolism such as forskolin (Jiraskova et al., 2018) which, in the present study, resulted in a non-physiological up-regulation of several genes (see Figures 5B,C). Taken together, these results indicate that placenta-derived carcinoma cells, BeWo, BeWo b30, and JEG-3, are not optimal *in vitro* models for TRP-related placental research; instead, use of primary human trophoblast cells is recommended.

Inconsistency exists in the current literature on IDO1 localization in the placenta and its expression in trophoblast cells (Sedlmayr et al., 2014). In the present study, we observed higher expression of *IDO1* in isolated CTB when compared to STB stage. While several studies report IDO1 in STB (Sedlmayr et al., 2002; Honig et al., 2004; Kudo et al., 2004) and CTB (Dong et al., 2008; Cvitic et al., 2013), recent papers (Ligam et al., 2005; Blaschitz et al., 2011) propose exclusive localization in vascular endothelium arguing that previous findings of IDO1 in trophoblast are a result of contaminating endothelial cells in isolated PHT cells (Sedlmayr et al., 2014). However, in our preparations, contamination with endothelial cells is routinely less than 1%, reflecting solely *IDO1* expression in CTBs. Moreover, for a long time it was believed that as pregnancy proceeds, the CTB layer gradually disappears (Benirschke et al., 2016); however, latest research reveals increasing number of CTBs at term (Mori et al., 2007) and designates them as the most metabolically active cells in human term placenta (Kolahi et al., 2017). Thus, for certain enzymes such as IDO1, CTB layer may be more active in metabolism than STB, and functional studies in isolated CTB cells should not be neglected.

**FIGURE 6:**
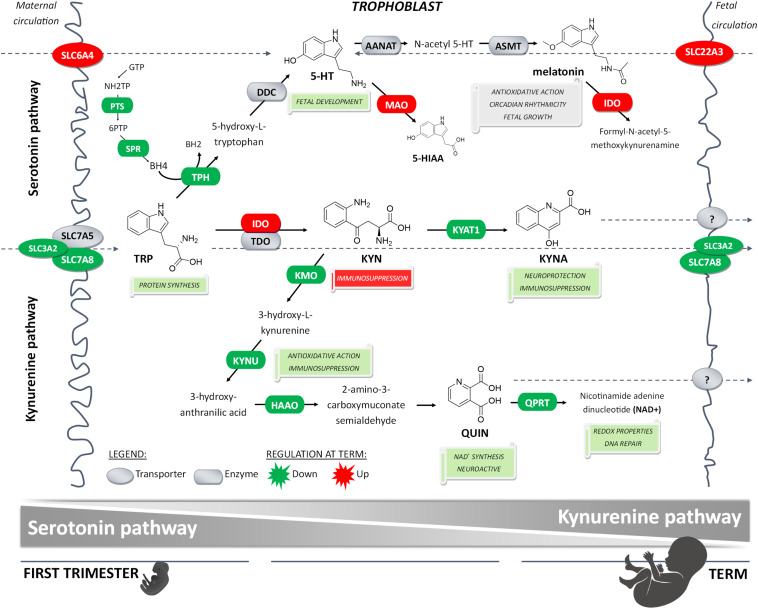
Proposed gestational age-dependent changes in placental TRP metabolic pathways. The schematic illustration is based on gene expression studies and, in the case of rate-limiting enzymes/key transporters, also on protein, and/or functional activity. The first-trimester placenta preferentially utilizes TRP for generation of 5-HT involved in fetal development and synthesis of KYN metabolites implicated with neuroprotection, antioxidative and redox effects, and DNA repair. On the other hand, the term placenta synthesizes significant amounts of KYN important for immune regulation activities. Collectively, our findings strongly indicate gestational age-related changes in placental handling of TRP and its main metabolites, 5-HT and KYN. This may reflect the changes in placental and fetal demands for metabolites of either pathway to ensure proper embryonic and fetal development throughout pregnancy.

In conclusion, here we report that placental homeostasis of TRP is subject to strictly regulated developmental changes during pregnancy (Figure 6). Considering the manifold role of TRP metabolites in placenta function, fetal development, and programming, tight regulation is necessary to maintain its homeostasis in the fetoplacental unit and ensure optimal communication on the placenta–brain axis. Subsequently, any internal or external insults, such as polymorphisms, epigenetics, pharmaceuticals or diseases, may compromise this harmonized interplay of enzymes and transporters, and result in suboptimal *in utero* conditions, and subsequently poor pregnancy outcomes. Importantly, timing of these insults is critical for fetal development (Barker et al., 2010); thus, knowledge of TRP catabolic pathways in the placenta during pregnancy aids in understanding the biological roots of fetal programming.

## Data Availability Statement

The original contributions presented in the study are included in the article/Supplementary Material, further inquiries can be directed to the corresponding author.

## Ethics Statement

The studies involving human participants were reviewed and approved by the University Hospital Research Ethics Committee (201006 S15P) and the Ethics Committee of the Canton of Bern (Basec No. 2016-00250). The participants provided their written informed consent to participate in this study.

## Author Contributions

RKa, CAb, and FS participated in the study concept and design. RKa, CAb, HH, PK, and JZ participated in the data acquisition. RKa, CAb, HH, PK, LC, RKu, CAl, and FS performed the data analysis and participated in interpretation of the results. RKa, CAb, and FS wrote the article. All authors contributed to the article and approved the submitted version.

## Conflict of Interest

The authors declare that the research was conducted in the absence of any commercial or financial relationships that could be construed as a potential conflict of interest.

## Footnotes

1 http://www.heatmapper.ca/
