# Supplementary material for: Dynamics of Tryptophan Metabolic Pathways in Human Placenta and Placental-Derived Cells: Effect of Gestation Age and Trophoblast Differentiation
Source: Front Cell Dev Biol. 2020 Sep 18;8:574034. doi: 10.3389/fcell.2020.574034 (PMC7530341; doi:10.3389/fcell.2020.574034)
Supplement: Supplementary file 1 [file Data_Sheet_1.PDF]

Supplementary Material

1 Supplementary Tables

Supplementary Table 1: List of predesigned probe assays analyzed by qPCR and ddPCR.

Gene symbol	Gene name	Gene alias	Assay ID
**FAM Probe**			
IDO1	indoleamine 2,3-dioxygenase 1	IDO-1	Hs00984148_m1
IDO2	indoleamine 2,3-dioxygenase 2	IDO-2	Hs01589373_m1
TDO2	tryptophan 2,3-dioxygenase	TDO	Hs00194611_m1
KMO	kynurenine 3-monooxygenase	-	Hs00175738_m1
KYAT1	kynurenine aminotransferase 1	CCBL1	Hs00187858_m1
KYNU	kynureninase	-	Hs01114105_m1
HAAO	3-hydroxyanthranilate 3,4-dioxygenase	3-HAO	Hs00895710_m1
QPRT	quinolinate phosphoribosyltransferase	QPRTase	Hs01547534_g1
TPH1	tryptophan hydroxylase 1	TRPH	Hs00188220_m1
TPH2	tryptophan hydroxylase 2	NTPH	Hs00542783_m1
PTS	6-pyruvoyltetrahydropterin synthase	PTPS	Hs00609393_m1
SPR	sepiapterin reductase	-	Hs00268403_m1
MAO-A	monoamine oxidase A	MAO-A	Hs00165140_m1
MAO-B	monoamine oxidase B	-	Hs01106246_m1
AANAT	aralkylamine N-acetyltransferase	SNAT	Hs01063208_g1
ASMT	acetylserotonin O-methyltransferase	ASMTY	Hs00187839_m1
SLC3A2	solute carrier family 3 member 2	4F2HC	Hs00374243_m1
SLC6A4	solute carrier family 6 member 4	SERT	Hs00984349_m1
SLC7A5	solute carrier family 7 member 5	LAT1	Hs01001189_m1
SLC7A8	solute carrier family 7 member 8	LAT2	Hs00794796_m1
SLC22A3	solute carrier family 22 member 3	OCT3	Hs01009571_m1
TBP	TATA-box binding protein	GTF2D	Hs00427620_m1
YWHAZ	tyrosine 3-monooxygenase/tryptophan 5-monooxygenase activation protein zeta	14-3-3-zeta	Hs01122445_g1

Gene symbol	Gene name	Gene alias	Assay ID
**HEX Probe**			
B2M	beta-2-microglobulin	IMD43	dHsaCPE5053101

TaqMan® FAM probe assays were obtained from Thermo Fisher Scientific, Waltham, MA, USA whereas HEX probe assay was obtained from BioRad, Hercules, CA, USA.

Supplementary Table 2: List of antibodies used in Western Blot analysis.

Protein	Product name	Distributor	Cat. No.	Dilution
**Target proteins**				
**SLC6A4**	Rabbit anti-serotonin transporter antibody	Abcam	ab181034	1:500
**SLC22A3**	Rabbit anti-SLC22A3/OCT3 antibody	Abcam	ab124826	1:10000
**MAO-A**	Rabbit anti-monoamine oxidase A antibody	Abcam	ab126751	1:1000
**IDO1**	Rabbit anti-IDO1 antibody	Thermo Fisher Scientific	PA5-79437	1:1000
**TPH1**	Rabbit anti-TPH1 antibody	Thermo Fisher Scientific	PA1-777	1:500
Secondary antibody	Swine anti-rabbit Immunoglobulins/HRP	Dako	P0217	1:10000
**Reference protein**				
**β-actin**	Mouse anti-beta actin antibody	Abcam	ab8226	1:10000
Secondary antibody	Rabbit anti-mouse Immunoglobulins/HRP	Dako	P0260	1:10000

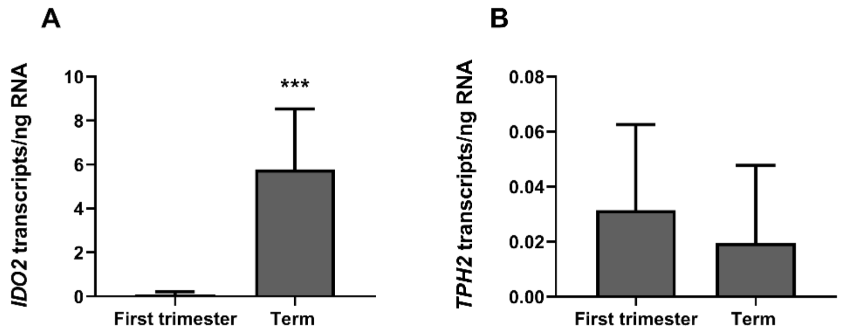

Supplementary Figure 1: Transcript levels of *TPH2* (A) and *IDO2* (B) in human first trimester and term placenta. Data are presented as mean ± SD and statistical significance was evaluated using unpaired t-tests; *** ($p \leq 0.001$).
